# Retroperitoneal Ewing's sarcoma/embryonal tumor: a rare differential diagnosis of back pain

**DOI:** 10.1590/0100-3984.2015.0236

**Published:** 2017

**Authors:** Fabiano Reis, Eduardo Macedo, Marcondes Cavalcanti França Junior, Eliane Ingrid Amstalden, Simone Appenzeller

**Affiliations:** 1 Universidade Estadual de Campinas (Unicamp), Campinas, SP, Brazil

**Figure 1:**
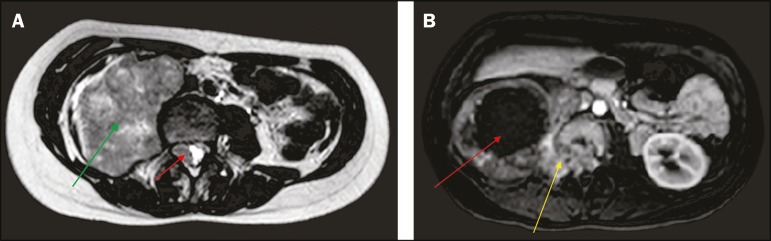
**A:** Axial T2-weighted MRI scan showing a huge heterogeneous retroperitoneal mass (green arrow) with an isointense intradural component (red arrow). **B:** Axial gadolinium contrast-enhanced T1-weighted image showing a large mass, with intense peripheral enhancement and central necrosis (red arrow), extending through the neural foramen (yellow arrow).

**Figure 2:**
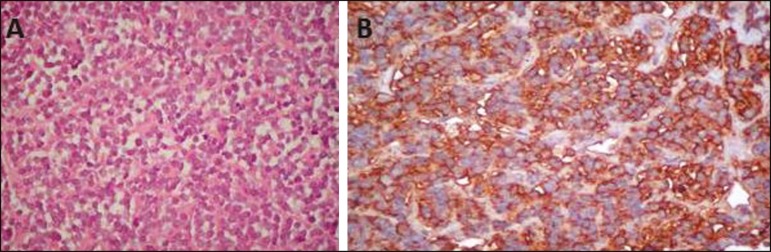
**A:** Hematoxylin-eosin staining (original magnification, ×40) showing dense cellular proliferation in a diffuse or vaguely lobular pattern of uniform round cells, with scanty cytoplasm, ovoid nuclei with fine chromatin and small nucleoli. Note also the delicate fibrous vascular stroma. **B:** CD99 (MIC-2) staining showing strong immunoreactivity of the cytoplasmic membrane.

*Dear Editor*,

A previous healthy 17-year-old female was referred to a rheumatology clinic due to a 6-month history of lower back pain. Her pain worsened at night and did not radiate. During that 6-month period, she had lost weight (5 kg). An initial evaluation produced normal cardiovascular and abdominal findings. She had pain on lumbar spine palpation and pain when her sacroiliac joints were examined (Patrick's test). Laboratory tests showed normal blood smear results and normal levels of inflammatory markers. While waiting for a magnetic resonance imaging (MRI) scan of her sacroiliac joint, she returned with significant worsening of her pain and weakness in her right leg. Examination showed grade 3 muscle strength and an absence of the ipsilateral patellar reflex. MRI revealed a right paravertebral mass, with intradural and foraminal components, showing a signal that was, in comparison with the muscle signal, predominantly isointense (with a hyperintense component indicating hemorrhage) on T1-weighted images and isointense (with a hyperintense necrotic component) on T2-weighted images (Figure 1). Ultrasound-guided biopsy revealed an undifferentiated small round-cell morphology. Immunohistochemistry staining suggested a member of the Ewing's sarcoma/embryonal tumor (ES/ET) family (Figure 2). The patient was submitted to chemotherapy, which did not elicit an adequate response.

ES/ETs belong to a rare group of malignant neoplasms with small round-cell morphology. Although these tumors arise from a common precursor cell, each entity represents a different expression of the same neoplasm, characterized by distinct cellular differentiation or anatomic location^(1)^.

The majority of ES/ETs are diagnosed during the first two decades of life^(1)^. The most common soft tissue sites are the chest wall, lower extremities, and pelvis/hip region. They are rarely found in the retroperitoneum, upper extremities, or internal organs^(2)^. Patients often present with a painless mass or vague abdominal or chest pain, depending on tumor site^(1)^. Muscle weakness can also be the predominant symptom^(3)^.

There are certain red flags that should always be considered in the differential diagnosis of patients with focal neurological manifestations of myelopathy and radiculopathy. Although rare and having no specific radiological findings, ES/ET should be suspected in young adults presenting with a large heterogeneous mass in the trunk, extremities, or soft tissues^(4)^. In the case presented here, an MRI finding of a large mass with isointense solid components on T1- and T2-weighted images, together with necrosis and hemorrhage, facilitated the diagnosis in this intriguing case. In addition, ES of the retroperitoneum is difficult to differentiate from other tumors. The retroperitoneal tumors that can invade the neural foramen and vertebral canal are the following: ganglioneuroma and ganglioneuroblastoma; neuroblastoma in younger patients (mean age, 22 months); leukemia (chloroma); and lymphoma. Invasion of the renal vein, inferior vena cava, and liver can be seen in ES, renal cell carcinoma, and adrenocortical carcinoma. Differentiation aspects that favor the diagnosis of ES are earlier age of presentation, absence of metastatic lymphadenopathy, and absence of calcifications. ES tends to be unilateral and does not cross midline^(5)^. The definitive diagnosis can be made only by histopathological analysis.
